# Semantic Feature Training in Combination with Transcranial Direct Current Stimulation (tDCS) for Progressive Anomia

**DOI:** 10.3389/fnhum.2017.00253

**Published:** 2017-05-16

**Authors:** Jinyi Hung, Ashley Bauer, Murray Grossman, Roy H. Hamilton, H. B. Coslett, Jamie Reilly

**Affiliations:** ^1^Eleanor M. Saffran Center for Cognitive Neuroscience, Temple University, PhiladelphiaPA, USA; ^2^Department of Communication Sciences and Disorders, Temple University, PhiladelphiaPA, USA; ^3^Penn Frontotemporal Degeneration Center, Department of Neurology, Perelman School of Medicine, University of Pennsylvania, PhiladelphiaPA, USA; ^4^Center for Cognitive Neuroscience, Department of Neurology, Perelman School of Medicine, University of Pennsylvania, PhiladelphiaPA, USA

**Keywords:** transcranial direct current stimulation (tDCS), language rehabilitation, primary progressive aphasia, semantic feature analysis, naming therapy

## Abstract

We examined the effectiveness of a 2-week regimen of a semantic feature training in combination with transcranial direct current stimulation (tDCS) for progressive naming impairment associated with primary progressive aphasia (*N* = 4) or early onset Alzheimer’s Disease (*N* = 1). Patients received a 2-week regimen (10 sessions) of anodal tDCS delivered over the left temporoparietal cortex while completing a language therapy that consisted of repeated naming and semantic feature generation. Therapy targets consisted of familiar people, household items, clothes, foods, places, hygiene implements, and activities. Untrained items from each semantic category provided item level controls. We analyzed naming accuracies at multiple timepoints (i.e., pre-, post-, 6-month follow-up) via a mixed effects logistic regression and individual differences in treatment responsiveness using a series of non-parametric McNemar tests. Patients showed advantages for naming trained over untrained items. These gains were evident immediately post tDCS. Trained items also showed a shallower rate of decline over 6-months relative to untrained items that showed continued progressive decline. Patients tolerated stimulation well, and sustained improvements in naming accuracy suggest that the current intervention approach is viable. Future implementation of a sham control condition will be crucial toward ascertaining whether neurostimulation and behavioral treatment act synergistically or alternatively whether treatment gains are exclusively attributable to either tDCS or the behavioral intervention.

## Introduction

Language is a complex, multifactorial construct that involves the precise orchestration of numerous functions (e.g., syntax, semantics, morphology, phonology). Acquired language disorders may emerge in the context of focal lesions that disrupt one or more of these systems or through damage to a supporting cast of supra-linguistic functions (e.g., working memory, visual perception) that support our ability to flexibly use language. Primary progressive aphasia (PPA) is a neurodegenerative condition characterized by an insidious loss of the ability to comprehend and/or produce language in the absence of generalized dementia (Mesulam, 1982, 2003). PPA is a phenotypic diagnosis based on the overt physical manifestation of an underlying disease process. The most common etiologies that produce PPA include frontotemporal degeneration or Alzheimer’s Disease (Mesulam et al., 2008; Grossman, 2010; Harciarek and Kertesz, 2011). In both of these neuropathologies, abnormal protein depositions result in neuronal dropout and macroscale atrophy within cortical regions that are critical for producing and/or perceiving language. The specific profile of language loss incurred in PPA is linked to the underlying anatomical distribution of cortical atrophy. Speech and language functions tend to be strongly left hemisphere lateralized (Price, 2010). Accordingly, when disease processes compromise the left hemisphere perisylvian language network, people typically experience a range of cognitive-linguistic impairments (Newhart et al., 2007; Grossman et al., 2013; Henry et al., 2016).

Language impairment, especially progressive anomia, is not only the prominent feature of PPA, but also one of the most commonly observed symptoms in the canonical presentation of Alzheimer’s Disease (AD) (Reilly et al., 2011).^1^ In the exploratory treatment study described below, we examined a cohort of patients, all of whom experience progressive naming impairments associated either with a fluent variant of PPA (semantic or logopenic variants of PPA) or early onset AD. We hereafter collectively refer to this worsening impairment for naming common objects and people as *progressive anomia*.

The question of how to promote language recovery in the context of progressive anomia is inherently complex, and such treatments are in their relative infancy. One might logically look to the more mature discipline of post-stroke aphasia rehabilitation as a reasonable starting point for designing therapies based on intrinsic (e.g., individual differences) and extrinsic (e.g., therapy dose and targeted cognitive function) factors that promote functional recovery. Unfortunately, there are many caveats associated with extrapolating from the known (i.e., post-stroke aphasia) to the relative unknown (i.e., PPA or dementia). During its chronic stage, post-stroke aphasia typically represents either static or improving conditions. In stark contrast, the profile of language loss in progressive anomia is neither static nor improving. Language in progressive anomia progressively worsens, and the rate and qualitative characteristics of lexical dropout (i.e., which words will be forgotten next) are unpredictable (Reilly, 2016). Progressive anomia, therefore, presents a moving target for rehabilitation paradigms whose traditional goal is restoration of a particular function. Reilly et al. (2005) and others (Jokel et al., 2014) have argued that the restorative approach of re-training forgotten words *ad hoc* has serious weaknesses and that maintenance of known words provides a more realistic and proactive substrate for treatment.

### Treatment Adjuvants for PPA

Non-invasive brain stimulation offers a potential therapeutic advance for the treatment of PPA. Transcranial direct current stimulation (tDCS) is one particular form of brain stimulation with numerous advantages (e.g., cost, portability, comfort) in terms of its clinical application to PPA. In conventional tDCS, a low amperage current typically ranging between 1 and 2 mA is applied to the scalp using probabilistic anatomical landmarking (10/20 EEG system) to guide the strategic placement of two or more electrode sponges (i.e., montages). The head (including skull, cerebrospinal fluid, air-filled sinuses and brain) effectively closes the circuit between anodal and cathodal electrode leads. Depending on the location of the electrodes, that is the montage, electrons flowing from cathode to anode will alter cell membrane potentials of specific groups of neurons (Nitsche and Paulus, 2000). Effects of tDCS are evident across a variety of timescales ranging from an hour or more to months after stimulation. Although the dynamics of neuroplastic change induced by tDCS remain poorly understood, it appears that dose (duration and current intensity) is a powerful moderating variable (Reinhart et al., 2016). In general, it has been argued that a longer stimulation protocol distributed over multiple sessions with higher current intensity is more likely to produce durable treatment effects (Nitsche and Paulus, 2000) (also see Reinhart et al., 2016 for other factors determining the tDCS effects). It is believed that anodal stimulation produces excitability in a swath of cortex proximal underlying the position of anodal lead(s) (Nitsche and Paulus, 2000; Cuypers et al., 2013).

The ability to selectively target and optimize membrane potentials within local populations of neurons is particularly relevant to the treatment of circumscribed brain injuries. In post-stroke aphasia, for example, many investigators believe that optimal recovery of language occurs on the context of peri/ipsilesional functional reorganization (Benjamin et al., 2014; Reilly et al., 2014). That is, recovery is best when the tissue surrounding a lesion assumes function of the damaged region. tDCS may prove useful in artificially stimulating tissue surrounding a lesion (Fridriksson et al., 2011; Richardson et al., 2015) or in either upregulating or suppressing the functioning of homologous regions of the contralateral hemisphere (Vines et al., 2011; Turkeltaub, 2015). In each of these cases, stimulation via a strategically placed distribution of electrodes may aid in improving language perception and/or production (Hamilton et al., 2011).

Few neurostimulation studies of AD have been reported to date, and this lack of precedence is a rate limiting factor in the optimization of electrode montage and dose. One potential target is the left temporoparietal region (Fernandez et al., 2002) that has previously shown hypoactivity (e.g., Schliebs and Arendt, 2011). Ferrucci et al. (2008) targeted the temporoparietal region and reported a positive effect of a one-session anodal stimulation on word recognition task (i.e., if a word was presented previously), while cathodal condition worsened the performance and the sham condition had no effect. Alternatively, anodal stimulation in the left DLPFC and temporal regions (i.e., site T7 in the 10/20 EEG system (Boggio et al., 2009) or sites T3 and T4 (Boggio et al., 2012)) also produced gains in a visual memory task. In terms of language function, previous work using non-invasive brain stimulation has shown that repetitive TMS or anodal tDCS stimulation to the DLPFC can potentially improve action and/or object naming performance in AD (Cotelli et al., 2008; Fertonani et al., 2010). Thus, though more investigation is needed, neurostimulation has the potential to confer benefits in the context of diffuse neuropathologies (e.g., AD) (also see Hansen, 2012).

Although tDCS does show promise as an independent therapy, a more compelling and potentially powerful application of this technology is as an adjuvant to behavioral treatment (Crinion, 2016). That is, tDCS paired with a behavioral treatment offers the promise of synergistic effects that would not otherwise be apparent through stimulation or behavioral treatment alone (Monti et al., 2013; Price et al., 2015; Tippett et al., 2015).

### Anomia in PPA and AD

Clinical criteria for PPA currently discriminate between three distinct variants (Gorno-Tempini et al., 2011). When cortical atrophy predominantly impacts the left posterior inferior frontal cortex, patients experience non-fluent/agrammatic PPA (nfvPPA), a syndrome characterized by slowed and imprecise speech output, along with agrammatism (Grossman, 2012). PPA also includes two variants with pathology that predominantly impacts the temporal lobes (i.e., logopenic variant PPA (lvPPA), and semantic variant PPA (svPPA)). Patients with lvPPA typically experience atrophy of posterior temporal and inferior parietal lobe structures and in turn present with a constellation of behavioral symptoms that include profound problems with sentence repetition. lvPPA naming difficulty has also been attributed to impaired lexical retrieval relative to frank semantic loss (Gorno-Tempini et al., 2008). In contrast, patients with svPPA experience profound impairments in word and object knowledge associated with the degeneration of ventral and anterolateral portions of the temporal lobes (Hodges and Patterson, 2007; Patterson et al., 2007; Bonner et al., 2016; Cousins et al., 2016). There seems to be relative preservation of knowledge of personal objects over the equivalent objects that are not used daily by the patient (e.g., a patient’s coffee cup versus any coffee cup) (Snowden et al., 1994; Bozeat et al., 2002; Giovannetti et al., 2006). It has been argued that the root cause of naming impairment in svPPA is the dimming of semantic memory, the substrate for word meaning.

Patients with Alzheimer’s Disease not explicitly linked to PPA also typically manifest naming impairments. However, the etiology of anomia in AD remains contested. Some have argued based on the presence of retained priming and cueing effects in AD (e.g., it has a tail, barks, and you walk it with a leash… it’s a_______) that its naming impairment reflects impaired access to knowledge (Reilly et al., 2011; Libon et al., 2013). Others have proposed that naming impairments in AD have a more fundamental root cause in degraded semantic knowledge (Hodges et al., 1992, 1996) or result from an interaction between degraded semantic knowledge and executive and perceptual processes that are crucial for lexical retrieval (Reilly et al., 2011). In the canonical presentation of AD, people experience plaque and tangle pathology that impacts a diffuse range of cortical subcortical regions (Holtzman et al., 2011). Previous neuroanatomical correlation studies do, however, demonstrate that plaque burden in AD is not uniformly distributed throughout the brain and that volumetric loss within particular regions (e.g., anterior temporal cortex (Frings et al., 2011; Domoto-Reilly et al., 2012) and lateral temporal cortex (Grossman et al., 2004)) is predictive of the severity of naming impairment. In neurotypical adults, it has been hypothesized these regions of the temporal lobe play crucial roles in conceptual representation (Reilly et al., 2016).

Speech and language deficits manifest differently across each of progressive aphasia and dementia variants (Reilly et al., 2010; Libon et al., 2013). Such variability precludes the application of a one-size-fits-all approach to language intervention and its formal assessment (e.g., Montembeault et al., 2016). For example, treatments targeting motor speech fluency are not readily applicable for patients with svPPA whose output is typically fluent. Likewise, re-training the conceptual attributes of a set of target words may prove ineffective for patients with nfvPPA who do not typically experience frank semantic impairments throughout the early stages of the disease. In the current study, we focused on treatment of progressive anomia associated with a semantically based naming impairment – progressive anomia – in a cohort of patients with fluent variant of PPA (svPPA or lvPPA) or AD.

### A Semantic Anomia Treatment Paired with tDCS

Reilly (2016) outlined both a treatment approach and item-selection protocol for semantic anomia (i.e., semantically based naming impairment) in svPPA. Briefly, this approach involves the maintenance of a small, carefully crafted micro-lexicon consisting of 100 words. The target items include a range of functional semantic categories, including familiar people, hygiene implements, foods, clothes, places, household items, and activities. When patients begin treatment, they are randomly assigned a set of training items from fixed lists, and items from these lists that are not assigned act as controls. For example, the assignment of clothes items for explicit training may include, “shirt, socks, glasses” while “pants, watch, hat” act as untrained stimuli. In this way, we impose item rather than participant level controls. In the first stage of the treatment, patients are assigned their target items in conjunction with the primary caregiver. We then travel to the patient’s home and take digital photographs of the trained and untrained items and array all the pictures within a book organized by semantic category.

Once a suitable item pool is established, the treatment involves acquisition of baseline neuropsychological measures followed by repeated training of the target lexicon using a modification of semantic feature analysis (SFA). SFA traditionally involves asking patients either to generate or verify a matrix of conceptual attributes (e.g., what is it? where is it found? what is it used for?) for a given stimulus (Boyle, 2004; Hashimoto and Frome, 2011). The goal of SFA, when applied to post-stroke aphasia, involves re-establishing weakened links between semantic knowledge that has come untethered to lexical representations. In the current treatment, we adapted this paradigm to an error-reduced format by reading the features aloud to the patient in blocks of five items and subsequently asking the patients to name the target item and generate the features he/she heard minutes before (Reilly, 2016). This treatment is then regularly repeated as disease severity worsens with the goal of maintaining a core functional vocabulary.

Here we examined the effectiveness of anodal tDCS administered to the left temporoparietal region as an adjuvant to this semantic treatment approach in a sample of patients with progressive anomia. The left temporoparietal region had previously reported as a pivotal hub region for semantic processing (Price et al., 2016; Reilly et al., 2016). We hypothesize that targeted stimulation of this region coupled with a behavioral semantic challenge will produce synergistic and potentially lasting gains that result in improved naming.

## Materials and Methods

To follow, we report the initial leg of an ongoing longitudinal treatment study. Patients (*N* = 5) completed a regimen of ten days of modified semantic feature analysis therapy paired with online anodal stimulation via tDCS. We evaluated the effects of treatment using a pre/post design with a 6-month follow-up to assess treatment maintenance.

### Participants

Participants (*N* = 5) included four individuals (3 males, ages 55–74, mean age = 66.6, SD = 8.56) diagnosed with a fluent variant of PPA (svPPA or lvPPA) and one individual with severe anomia associated with early onset AD. Diagnoses were made by experienced behavioral neurologists (i.e., Grossman, Coslett, Hamilton) at the University of Pennsylvania in accord with published diagnostic criteria (Gorno-Tempini et al., 2011; McKhann et al., 2011). All participants were right-handed, native speakers of English with no history of seizures, implanted medical devices, or previous brain injuries (e.g., stroke). All participants gave informed consent and the study was approved by the Institutional Review Board of the University of Pennsylvania. **Table 1** reflects neuropsychological and demographic characteristics of the patient sample.

**Table 1:** Demographic and neuropsychological data.

						MoCA			BNT			PPT-p/w			Trail a/b			Digits b/f			Fluency f		
ID	Age	Sex	Dx	Edu	Y_Post_	*T_1_*	*T_2_*	*T_3_*	*T_1_*	*T_2_*	*T_3_*	*T_1_*	*T_2_*	*T_3_*	*T_1_*	*T_2_*	*T_3_*	*T_1_*	*T_2_*	*T_3_*	*T_1_*	*T_2_*	*T_3_*
CM	71	F	lvPPA	16	3	18	20	18	6	4	5	25/ 24	25/ 25	23/ 23	45/ 131	32/ 139	33/ 177	3/4	3/3	4/2	8	8	8
MC	60	M	AD	18	4	U	U	U	6	7	4	10/ U	13/ 16	12/ 11	295/ 300	300/ 300	544/ U	2/5	1/2	1/4	5	6	U
VM	74	M	svPPA	18	4	14	12	7	1	1	1	23/ 17	18/ 16	16/ 19	37/ 101	34/ 95	35/ 117	5/4	6/5	5/6	8	8	5
JB	55	F	svPPA/ bvFTD	18	2	16	22	13	1	2	1	18/ 16	18/ 16	16/13	39/ 63	39/ 63	48/122	8/8	11/9	10/8	5	2	2
RC	73	M	svPPA	22	5	11	15	15	1	1	0	16/ 15	16/ 13	13/ 11	51/ 105	43/ 101	52/ 184	6/5	6/7	7/6	8	9	5

*U = verbally refused task; Dx = Diagnosis; Edu = Education in years; Test stage = reflected testing time at pre-stimulation (T_*1*_), post-stimulation (T_*2*_), and 6 month follow-up (T_*3*_); Y_Post_ = number of years from the onset year of the self-report behavioral and cognitive issues to behavioral treatment onset; MoCA = Montreal Cognitive Assessment (Nasreddine et al., 2005); BNT = Boston Naming Test (short form; *N* = 15) (del Toro et al., 2011); PPT-p/w = Pyramids and Palm Trees Test picture and word version, short form with total raw score of 26 (Howard and Patterson, 1992); Trails a/b (in second) = the amount of time spent in connecting trails (Reitan, 1992); Digits f/b = Digit span forward and backward from the Wechsler digit span subtest (Wechsler, 2008); Fluency F = the number of valid words beginning with letter F generated in a minute; lvPPA = Logopenic variant primary progressive aphasia; svPPA = semantic variant primary progressive aphasia; bvFTD = behavioral variant frontotemporal degeneration; AD = Alzheimer’s disease.*

### Materials

We tailored a target lexicon consisting of approximately 100 words to each patient (see Reilly, 2016 for detailed selection criteria). Briefly, target items were quasi-randomly selected from fixed lists representing the following six semantic categories: places, foods, clothes, household items, activities, and hygiene implements. Items in the fixed lists were normed by a group of healthy older adults (*N* = 15, mean age = 67.7) who were not involved in the study. Low frequency or familiar items were excluded and thus the fixed list contained 30 comparable items in each category (mean frequency = 4.84, mean familiarity = 4.86 on a 7-point Likert scale).

At baseline, we assigned training items by first selecting 15 target words from lists that consisted of 30 items per category (e.g., 15 articles of clothing). Unassigned target words from each semantic category served as item-level controls throughout the treatment. In some cases, patients were assigned items that had little functional utility (e.g., subject CM never wears ties), and for these cases we permitted the patient and caregiver together to swap in new items from the original list.^2^ After assignment, we compared the word frequency and familiarity between trained and untrained items for each subject. The subjective frequency and familiarity ratings (i.e., the degree to which one comes into contact with a word’s concept) were not very different between trained and untrained items for subject CM and RC. Although statistical significance showed difference for the other three patients, the difference was less than 0.6 units on a 7-point Likert scale indicating small effects. We also included a category of familiar people (*N* = 15) with the constraint that patients and caregivers should work together to include people who are frequently encountered, highly familiar, and have high personal relevance (e.g., a spouse or caregiver but not a grandchild seen once a year). For the ‘people’ category, it was therefore impossible to randomly assign a set of target items. For the people training condition, we included a set of famous faces (*N* = 15) as item-level controls.

Once a suitable item pool was established for each patient, we traveled to that patient’s home and obtained digital photographs of as many of the trained and untrained items as possible. We then generated a communication book/binder for each participant by arraying 500 pixel^2^ photographs in groups of 4 per-page clustered by semantic category (e.g., people, foods, hygiene implements, etc.).

### Treatment and Assessment Procedures

Semantic feature analysis typically involves asking the patient to either generate or verify a set of features for a given target item (Boyle, 2004; Hashimoto and Frome, 2011). We modified these task demands to capitalize on the benefit of an error-reduced learning approach (Frattali, 2004; Jokel and Anderson, 2012). We did so by having the treating clinician first announce the name of the target item and identify five of its constituent semantic features. We employed distinct semantic feature matrices for people and objects. For objects, features included use (i.e., what do you do with it?), physical appearance (i.e., what does it look like?), location (i.e., where do you find this?), and association (i.e., what is one thing that you often see near/with this?). For people, the matrices included relation (i.e., how do you know the person?), physical appearance, activity (i.e., what do you do together?), and fact (i.e., what’s an interesting fact about the person?).

For the instruction component, the clinician announced the name and features for blocks of five items. Immediately afterward, we cued the patient to name, generate features, and produce a novel sentence for the items that he/she just reviewed. Patients were instructed to avoid empty statements (e.g., an apple is a thing.). Using this technique of explicit instruction coupled with self-generation, we repeatedly cycled through the item pool.

### Neurostimulation Parameters

Patients completed ten days of tDCS paired with behavioral therapy spaced over two consecutive weeks (i.e., Monday–Friday). Each tDCS session began by seating the patient and probabilistically landmarking the left temporoparietal region on the scalp using the 10/20 EEG coordinate system (Nitsche et al., 2008). We placed the anode at the left P3, and the cathode was centered over the forehead. The 1 cm^2^ rubber electrode leads were placed within 5 cm^2^ sponges that were saturated with saline and fixed in place on the scalp using rubber straps. Once the electrodes were placed, we administered tDCS using a Magstim Eldith 1 Channel DC Stimulator. Patients received 20 minutes of 1.5 mA with ramp up/down periods of 30 s.

### Procedure and Design

We employed a pre/post treatment design pairing the behavioral intervention with online anodal tDCS. Prior to initiating the treatment, we administered a battery of neuropsychological assessments and gauged baseline naming for both the trained and untrained items. We conducted post-testing immediately after the treatment (i.e., at 2 weeks) with follow-up testing at 6 months. During each treatment session, we initiated behavioral treatment within 5 min of the onset of tDCS stimulation, and the behavioral treatment was administered for a duration of approximately 30 min per session (extending 5–10 min past tDCS rampdown to finish the behavioral intervention in a session).

Treatment consisted of a clinician (author AB) announcing the name of each target word followed by an explicit description of its semantic features. The patient was then cued to name each word, self-generate its features and generate a novel sentence containing the target word. Responses were considered correct if the target name was successfully produced. Patients were trained on 21 target items each day, 3 from each category, repeated twice through 10 sessions.

### Statistical Analysis

We analyzed accuracy data using the R statistical software package lme4 (Bates et al., 2015) via logistic mixed effects model. We treated individual patients and target items as random factors (Baayen et al., 2008; Jaeger, 2008) and entered the following as fixed factors in the model: Time (three levels, pre-stimulation, post-stimulation, 6-month follow-up), Item type (two levels, trained, untrained), and item category (seven levels, activity, clothes, food, household, hygiene, people, place). Individual participant data were analyzed via McNemar’s tests.

## Results

### Group Treatment Effects

**FIGURE 1:**
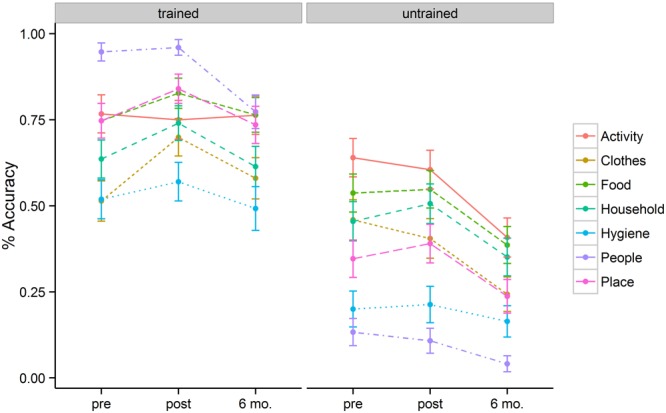
**Mean percentage of correct responses for each category, separated for the trained and untrained items, at pre-, post-stimulation, and 6 month follow-up**.

**Figure 1** represents a plot of accuracy data across semantic categories for the trained and untrained items at three different time points (baseline, immediately post tDCS, and at 6-month follow-up). The best fitting model revealed three significant factors: Time, Item type, and Semantic Category.

The baseline naming accuracy was 69% for trained items and 40% for untrained items (*p* < 0.001). Although trained and untrained item lists were comparable in general item frequency and familiarity, the higher performance for the trained items at baseline could be related to the nature of functional utility to patients themselves. After 2 weeks of behavioral + tDCS treatment, patients showed accuracies of 77% for trained and 41% for untrained items. Six months post treatment, patients showed accuracies of 68% for trained items and 27% for untrained items. These accuracy differences resulted in a significant effect of training (*b* = 1.54, *SE* = 0.18, *p* < 0.001). The magnitude of the treatment effect diminished at 6-month follow-up (*b* = –0.97, *SE* = 0.18, *p* < 0.001). Patients persisted in naming trained items more accurately than untrained items at post stimulation (*b* = 0.56, *SE* = 0.23, *p* = 0.02) as well as at 6-month follow-up (*b* = 0.73, *SE* = 0.24, *p* = 0.002).

**FIGURE 2:**
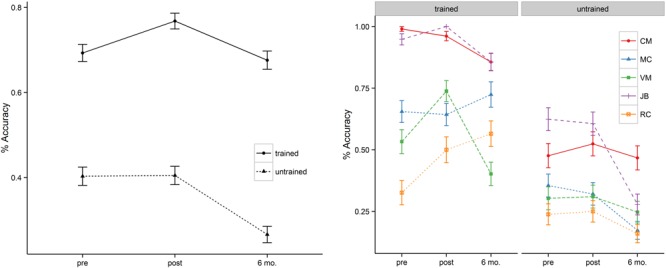
**Mean percentage of correct responses at pre-, post-stimulation, and 6 month follow-up (left = group; right = individual).** MC’s naming accuracy of the trained items at 6 month follow-up reflects the average of a reduced naming item list (also see the note in **Table 3**).

In addition to the main effect of treatment and time, patients showed a significant interaction between these two variables. This interaction was such that for trained items there was significant improvement in naming immediately post tDCS (*b* = 0.63, *SE* = 0.18, *p* < 0.001) but that these improvements fell to near baseline levels at the 6-month follow-up (69% vs. 68%). Untrained items, however, showed a flat pattern of response accuracy from pre- to post-tDCS (40% vs. 41%) over a 2 week span. Moreover, the untrained items showed significant drop-off at 6-month follow-up relative to the pre-treatment baseline (*b* = –0.96, *SE* = 0.16, *p* < 0.001). **Figure 2** (left panel) illustrates the relevant main effects and interactions.

The magnitude of the treatment effects differed across semantic categories at different time points. To better understand the interaction, we conducted individual mixed effect models to examine the effects of Time for each semantic category. Trained items were more likely to show positive improvement immediately after tDCS. The most prominent improvements were seen in naming accuracy for inanimate object categories such as Clothes (51% → 70%, *p* = 0.007), Household Items (64% → 74%, *p* = 0.07), and Places (75% → 84%, trend, *p* = 0.094). However, untrained items did not show significant improvement post tDCS, indicating that treatment gains did not diffuse within semantic categories (e.g., improvements in naming of ‘socks’ did not see parallel improvements in naming ‘shirts’). At the 6 month follow-up, patients showed marked loss in the naming of several semantic categories. Among trained items, patients performed worse at 6-months for Hygiene (*p* = 0.04) and People (*p* = 0.003). Among untrained items, patients showed significant decrements in naming accuracy at 6-months for most of the categories: Activities (*p* = 0.002), Clothes (*p* < 0.001), Food (*p* = 0.01), Household (trend, *p* = 0.098), Place (*p* = 0.05), and People (*p* = 0.05). **Table 2** reflects the mean accuracy for each semantic category.

**Table 2:** Mean accuracy for each category.

	Time_pre_	Time_post_	Time_6 month_
**Trained item**			
Activity	0.77	0.75	0.76
Clothes	0.51	0.70^∗∗^	0.58
Food	0.75	0.83	0.76
Household	0.64	0.74ˆ	0.61
Hygiene	0.52	0.57	0.49^∗^
People	0.95	0.96	0.77^∗∗^
Place	0.75	0.84ˆ	0.74
**Untrained items**			
Activity	0.64	0.61	0.41^∗∗^
Clothes	0.46	0.41	0.24^∗∗∗^
Food	0.54	0.55	0.39^∗^
Household	0.46	0.51	0.35ˆ
Hygiene	0.20	0.21	0.16
People	0.13	0.11	0.04^∗^
Place	0.35	0.39	0.24^∗^

*We reported statistical significance based on logistic mixed effect models. Formula: glmer (Accuracy ∼ Time + (1 | Subject) + (1 | Item), data, family = “binomial”).*

*Significance codes: ^∗∗∗^<0.001, ^∗∗^<0.01, ^∗^<0.05, ˆ∼0.05*

### Individual Outcomes

We assessed naming accuracy at three discrete time points (pre-, post-, 6-months) within subjects. **Table 3** reflects individual accuracies at each of these time points. This design yields three potential pairwise comparisons (T_1_–T_2_, T_1_–T_3_, and T_2_–T_3_). We derived accuracy change scores within participants using the following formula: (Mean_Post_–Mean_Pre_)/Mean_Pre_. In the derivation of these difference scores, “pre” represents the baseline for each comparison. That is, observation T_1_ is “pre” for the contrast of T_1_–T_2_ and T_1_–T_3_, whereas observation T_2_ is “pre” for the contrast of T_2_–T_3_. The extent of improvement between time points was compared using non-parametric, paired sample McNemar’s tests.

**Table 3:** Number of correct response at the pre-, post-stimulation, and 6-month follow-up for the trained and untrained items.

	CM			MC			VM			JB			RC		
Category	*T_1_*	*T_2_*	*T_3_*	*T_1_*	*T_2_*	*T_3_*	*T_1_*	*T_2_*	*T_3_*	*T_1_*	*T_2_*	*T_3_*	*T_1_*	*T_2_*	*T_3_*
**Trained**															
Activity	15/15	14/15	15/15	13/15	10/15	10/14	8/15	10/15	11/15	na	na	12/15	10/15	11/15	9/15
Clothes	12/13	11/13	10/14	5/16	8/15	5/10	3/15	8/15	4/15	15/15	15/15	10/15	3/15	9/15	11/15
Food	15/15	14/15	13/15	12/15	13/15	11/12	10/15	12/15	6/15	15/15	15/15	15/15	4/15	8/15	10/15
Household	15/15	15/15	13/15	8/15	8/15	6/8	6/15	12/15	5/15	16/17	17/17	14/17	4/15	5/15	5/15
Hygiene	15/15	15/15	9/15	4/16	3/16	U	2/15	7/15	2/15	18/18	18/18	16/18	2/15	2/15	4/15
People	15/15	15/15	14/15	23/24	22/24	18/24	14/17	16/17	7/17	17/17	17/17	17/17	2/2	2/2	2/2
Place	15/15	15/15	15/15	11/15	10/15	5/8	14/15	14/15	8/15	11/15	15/15	11/15	5/15	9/15	11/15
Total	102/103	99/103	89/104	76/116	74/115	55/76	57/107	79/107	43/107	92/97	97/97	95/112	30/92	46/92	52/92
**Untrained**															
Activity	10/15	10/15	9/15	11/15	9/15	4/15	7/15	8/16	7/16	12/15	10/15	6/15	8/15	9/15	5/15
Clothes	9/15	9/15	7/15	5/16	2/16	1/17	9/14	8/14	5/13	8/14	10/14	4/14	3/15	1/15	1/15
Food	9/15	7/15	9/15	9/17	11/19	9/19	7/15	6/15	5/14	17/20	19/20	7/20	2/15	3/15	2/15
Household	8/15	9/15	9/15	5/17	5/17	3/17	3/15	4/15	4/15	14/15	14/15	6/15	5/15	7/15	5/15
Hygiene	3/15	6/15	5/15	1/9	2/10	1/11	1/10	1/10	1/9	5/14	4/14	1/14	2/12	0/12	0/12
People	3/15	4/15	2/15	5/15	3/14	1/14	0/15	0/15	0/15	2/15	1/15	0/15	0/15	0/15	0/15
Place	8/15	10/15	8/15	2/18	3/18	1/18	3/15	4/15	2/15	10/16	8/16	5/16	4/14	5/13	3/13
Total	50/105	55/105	49/105	38/107	35/109	20/111	30/99	31/100	24/97	68/109	66/109	29/109	24/101	25/100	16/100

*U = unavailable for testing; na = not tested; Test stage = reflected testing time at pre-stimulation (T_*1*_), post-stimulation (T_*2*_), and 6 month follow-up (T_*3*_). MC requested to reduce the number of his training items due to the progression of his disease. Therefore, we reported his naming performance based on the reduced item list at 6 month follow-up.*

Two patients (i.e., VM, RC) showed significant improvement in naming accuracy for trained items post-stimulation (T_1_–T_2_) with changes in accuracy ranging from 38 to 53%. Subject JB improved her naming accuracy to 100% at post stimulation but together with subject CM they maintained near ceiling performance (>95%) at post-stimulation and they demonstrated small naming accuracy decrement at 6 month follow-up (∼ 10%). Another participant (subject MC – early onset AD) showed no significant effects of treatment in a contrast of pre- and post-stimulation. Subject VM showed greater naming accuracy decrement at 6 month followup (T_1_–T_3_ = 25%) but only subject RC continued to show positive improvement (T_1_–T_3_ = 73%). Whereas the group as a whole showed no improvement for the untrained items at post-stimulation (T_1_–T_2_), subject MC (T_1_–T_3_ = –49%), JB (T_1_–T_3_ = –55%), and RC (T_2_–T_3_ = –36%) showed significant drop-off at 6 month follow-up. See **Table 3** and **Figure 1** (right panel) for the individual participant performance.

## General Discussion

In the burgeoning field of language rehabilitation for dementia, it has become clear that progressive disorders require a unique approach that considers the dynamic nature of the associated language impairment (Bier and Macoir, 2010; Savage et al., 2014; Jokel et al., 2016). We have proposed such an approach premised upon the maintenance of a carefully crafted lexicon with high functional relevance. There are many advantages to working with such a closed set of stimuli. Foremost, lexical dropout is unpredictable in dementia and an approach premised upon retraining newly forgotten words *ad hoc* is simply not feasible given the diversity and size of our lexicon. Moreover, it is likely more challenging to relearn and subsequently retain a forgotten word than it is to maintain a known word. There also may be some advantage to being guided by the relative preservation of knowledge of personal objects over the equivalent objects that are not used daily by the patient. In these respects, working with a constrained item pool offers a proactive approach toward protecting a core vocabulary as disease severity worsens.

Much remains to be learned about methods for optimizing treatment to promote maintenance and generalization of gains. Here we evaluated the synergistic effects of pairing a behavioral treatment (semantic feature analysis) with anodal stimulation via tDCS delivered over a pivotal region of the brain for semantic processing, both in terms of feature integration (Binder et al., 2009; Price et al., 2016) and executive processes linked to semantic control (Jefferies and Lambon Ralph, 2006). Patients tolerated the procedure well, and treatment gains were evident immediately post tDCS with maintenance at six-month follow-up. Of note, patients did not show evidence of generalization from trained to untrained items within semantic categories. Rather, gains in naming accuracy were limited to the treated target lexicon. In addition, the magnitude of the treatment effects was moderated by semantic category with the most robust gains for inanimate objects and the steepest pattern of loss for the names of familiar people.

Patients showed lasting benefits for naming trained items relative to a steeper sloping loss for untrained item-level controls. The behavioral treatment we employed capitalizes on the closed nature of the training set, its personal/contextual familiarity, and its functional nature in daily life. We did not observe generalization to untrained items or offline standardized naming assessments. However, this lack of generalization was not unanticipated. Reilly (2016) advocated abandoning the goal of generalization in the context of progressive anomia with targeted selection of context-specific knowledge (i.e., naming one’s own dog relative to the broader superordinate distinction of dogs).

The overall group results should be interpreted with caution when considering our small cohort and the impact of individual differences. For the trained items, two participants improved significantly at post-stimulation, whereas three patients remained stable pre/post stimulation. Of the three patients who showed no change pre/post, two patients performed at ceiling, whereas the other patient showed a pattern of leveling with fair accuracy. In contrast, all participants showed a stable pattern of naming difficulty for untrained items pre/post stimulation. The patients most likely to see a sustained benefit from this treatment (subject VM, RC) were all diagnosed with semantic variant PPA. In contrast, the patient who was least responsive to treatment (subject MC) was diagnosed with a rapidly progressive form of early onset Alzheimer’s disease.

One weakness of the study is its small sample size. Despite the potential for small sample bias, we are encouraged that these results provide proof of concept that the treatment is well-tolerated and show potential for benefit. Moreover, the sample size we employed is not unaligned with other contemporary patient-based tDCS studies. Marangolo et al. (2011) administered tDCS to three people with chronic aphasic and concomitant apraxia of speech. These patients received language treatment for five consecutive days pairing with anodal (20 min, 1 mA) and sham stimulation. Although patients showed improvement in both anodal and sham conditions post treatment in terms of speech articulation, the effect was greater in the anodal condition post tDCS and there was only retention in the anodal condition. Fiori et al. (2011) also investigated tDCS paired with behavioral treatment in chronic aphasia (*N* = 3). For the two patients who were able to attend the follow-up, the effects of tDCS on naming accuracy and speed persisted for 3 weeks after treatment. Among the very few tDCS studies with neurogenerative disease, Tsapkini et al. (2014) reported 6 nfvPPA and lvPPA patients following 3 weeks (15 sessions) of stimulation paired with a spelling intervention. Although patients’ spelling was improved in both tDCS and sham stimulation, generalization to the untrained items was only evident in the anodal condition and its effects persisted for a longer duration than the sham condition. The current study adds to this incipient body of treatment research, calling for further investigation.

Unlike most other past studies, we have not reported a sham condition in our current investigation (also see Hesse et al., 2007). Therefore, one cannot reliably discern whether tDCS and the behavioral treatment interacted synergistically to improve naming. Instead, the only conclusion(s) that can reasonably be made are that naming improved and that patients tolerated the procedure well. Thus, the question of whether anodal tDCS acted as a true adjuvant to the behavioral treatment remains unclear.

Although the majority of patients showed improvement, this was not the case with the single AD patient we treated. Both AD and svPPA patients experience progressive anomia as a result of lexical-semantic impairment. We hypothesized that our stimulation montage would upregulate the underlying semantic system in conjunction with a behavioral semantic treatment. The AD patient’s lack of treatment benefit has several possible bases. This patient has a rapidly progressive form of early onset AD and showed the most severe global cognitive impairment of the patient cohort, including profound deficits in sustained attention. Tsapkini et al. (2014) argued accordingly that treatment is likely optimized by intervening in the earliest stages of PPA.

Other limitations of the study include lack of random assignment to an alternative treatment and/or the lack of a control stimulation site to evaluate the effectiveness of the particular electrode montage. We aim to address these weaknesses via comparison of the current patient cohort to itself through behavioral treatment plus sham and a second patient cohort who will undergo sham followed by tDCS.

## Conclusion

The current study adds to an incipient, albeit growing body of research demonstrating the efficacy of pairing anodal tDCS with a behavioral language intervention in PPA (Cotelli et al., 2014; Tsapkini et al., 2014; Gervits et al., 2015). Our results suggest that language maintenance is indeed possible in the context of progressive anomia, and we can conclude that patients tolerated active stimulation well. However, we are limited by the lack of suitable control conditions for language intervention in combination with tDCS (e.g., sham stimulation or active stimulation of cortical regions outside the canonical semantic network). Future designs that implement randomized controls will be crucial toward demonstrating efficacy for larger scale clinical trials.

## Ethics Statement

This study has been reviewed and approved by the Institutional Review Board at Temple University (PI:JR) and at the University of Pennsylvania (PI: HC). Research technician (AB) explained the protocol and went through each page of the informed consent with participants and their caregivers. Research technician made sure that participants and their caregivers fully understood the protocol and their right to withdraw at any time. Research technician also made sure that participants and their caregivers had the contact information of the research team and they should not hesitate to contact the team if they had any questions or concerns.

## Author Contributions

JH, JR, RH, and AB prepared the manuscript. HC and MG also assisted with manuscript preparation and oversaw data collection. All of the authors assisted with the design of the study and approved the final version of the manuscript.

## Conflict of Interest Statement

The authors declare that the research was conducted in the absence of any commercial or financial relationships that could be construed as a potential conflict of interest.

## Footnotes

1 The distinction between histopathology and phenotype in PPA is currently an area of intense biomarker research. The logopenic variant of PPA (lvPPA) is most often linked to Alzheimer’s Disease pathology and is considered an atypical AD variant (Migliaccio et al., 2009; Gorno-Tempini et al., 2011). The most typical presentation of AD reflects a combination of amnestic, perceptual, and linguistic disturbances (Reilly et al., 2010).

2 We crafted male/female item lists, tailoring the categories of ‘clothes’ and ‘hygiene’ to the patients’ most likely preferences (e.g., bra or mascara for women).
